# The Impact of Digital Technology on Self-Management in Cancer: Systematic Review

**DOI:** 10.2196/45145

**Published:** 2023-11-22

**Authors:** Dwight Su Chun Lim, Benedict Kwok, Patricia Williams, Bogda Koczwara

**Affiliations:** 1 College of Medicine and Public Health Flinders University Adelaide Australia; 2 Flinders Digital Health Research Centre Flinders University Adelaide Australia; 3 Department of Medical Oncology Flinders Medical Centre Adelaide Australia

**Keywords:** self-management, self-management support, self-management core skills, digital technology, digital health, mHealth, mobile health, eHealth, cancer, theoretical frameworks, predictors of effect, chronic disease, skills, decision-making, cancer treatment

## Abstract

**Background:**

Self-management (SM) plays an important role in supporting patients’ adaptation to and management of the symptoms of chronic diseases. Cancer is a chronic disease that requires patients to have responsibility in management. Digital technology has the potential to enhance SM support, but there is little data on what SM skills are most commonly supported by digital technology.

**Objective:**

This review aimed to examine the SM core skills that were enabled and supported by digital interventions in people with cancer and identify any predictors of the effect of digital health intervention on SM core skills.

**Methods:**

Three electronic databases (MEDLINE, Scopus, and CINAHL) were searched for papers, published from January 2010 to February 2022, that reported randomized controlled trials (RCTs) involving patients with cancer or survivors of cancer where a digital technology intervention was evaluated and change in 1 or more SM core skills was a measured outcome.

**Results:**

This systematic review resulted in 12 studies that were eligible to identify which SM core skills were enabled and supported by digital intervention. The total number of participants in the 12 studies was 2627. The most common SM core skills targeted by interventions were decision-making, goal setting, and partnering with health professionals. A total of 8 (67%) out of 12 RCTs demonstrated statistically significant improvement in outcomes including self-efficacy, survivorship care knowledge and attitude, quality of life, increased knowledge of treatment, and emotional and social functioning. A total of 5 (62%) out of 8 positive RCTs used theoretical considerations in their study design; whereas in 1 (25%) out of 4 negative RCTs, theoretical considerations were used. In 3 studies, some factors were identified that were associated with the development of SM core skills, which included younger age (regression coefficient [RC]=–0.06, 95% CI –0.10 to –0.02; *P*=.002), computer literacy (RC=–0.20, 95% CI –0.37 to –0.03; *P*=.02), completing cancer treatment (Cohen *d*=0.31), male sex (SD 0.34 in social functioning; *P*=.009), higher education (SD 0.19 in social functioning; *P*=.04), and being a recipient of chemotherapy (SD 0.36 in depression; *P*=.008). In all 3 studies, there were no shared identical factors that supported the development of SM core skills, whereby each study had a unique set of factors that supported the development of SM core skills.

**Conclusions:**

Digital technology for patients with cancer appears to improve SM core skills including decision-making, goal setting, and partnering with health care partners. This effect is greater in people who are younger, male, educated, highly computer literate, completing cancer treatment, or a recipient of chemotherapy. Future research should focus on targeting multiple SM core skills and identifying predictors of the effect of digital technology intervention.

**Trial Registration:**

PROSPERO CRD42021221922; https://tinyurl.com/mrx3pfax

## Introduction

Self-management (SM) is defined as the ability of an individual to manage the symptoms, treatment, physical and psychosocial consequences, and lifestyle changes inherent in living with a chronic condition. It is an important component of the management of many chronic conditions, including cancer [1]. SM requires patients to apply specific skills such as problem-solving, decision-making, behavioral monitoring and tailoring, setting goals, partnering with health care providers, and using resources [1]. SM support (SMS) provided by the health care system is often necessary to enhance, enable, and support a patient’s SM and includes activities, interventions, or programs to promote the patient’s skill and confidence in managing their chronic condition [2].

Digital technology, where technological interventions seek to provide improved health care, is one of the means of delivering SMS for people with cancer [3]. It uses a variety of approaches including web-based education, telecommunication with health care providers, delivery of remote rehabilitation programs or monitoring, decision support, and reporting of symptoms [1]. Digital SMS can be provided through a variety of channels such as mobile phone apps, text messages, social media, websites, and wearable devices [4]. The advances in mHealth (mobile health) technology offer a promise of improvement in symptom management on treatment through better SM [5].

To date, there are several reviews on digital health technology interventions that involve SMS for people with cancer [6-12]. These reviews support emerging evidence for improved outcomes with a variety of digital technological interventions supporting SMS in patients with cancer. The focus of these reviews was mainly on patient outcomes such as pain, psychosocial outcomes, and sleep with less attention to specific components of SM. These specific components include (1) problem-solving, (2) decision-making, (3) behavioral self-monitoring and tailoring, (4) setting goals, (5) partnering with health care providers, and (6) risk reduction. In addition, no clear conclusion has been drawn from the reviews as to whether specific patients’ characteristics were associated with different outcomes. An exploration of potential predictors of effective SMS such as age, sex, or socioeconomic background could allow greater tailoring of digital technology. This highlights a gap in this literature on how digital technology can enable the specific components of SM and the patients’ characteristics may impact the effectiveness of building SM core skills.

A systematic review by Boulley et al [7] reviewed 29 papers from 2001 up to 2017 reporting on cancer-related digital interventions to examine their components, the elements of engagement with digital interventions, and the psychosocial variables targets in the context of SM. The results showed a high level of engagement with digital technology, where it was shown that self-efficacy, psychological symptoms, and quality of life were the most commonly assessed study outcomes. Considerable heterogeneity was noted in components of digital interventions and measures for their engagement [7]. The authors concluded that digital technology could be effective in helping patients cope with the disease but further research into intervention components and engagement was needed to have a greater understanding of the mechanisms underlying the psychological and behavioral changes of patients with cancer or survivors of cancer. They also noted that older patients had high acceptability toward modern and often unfamiliar technology in 3 studies, challenging the perception that modern technology was less likely to be used by older populations [13].

Hernandez Silva et al [6] reviewed 7 papers up to 2017 to assess how mHealth interventions (a subgroup of digital technology where health care interventions can be delivered via personal mobile phone apps) could be used to improve pain, psychological distress, fatigue, or sleep outcomes on a heterogeneous population of survivors of cancer by supporting SM. A total of 3 (75%) out of 4 studies showed improvement in pain and 2 demonstrated improvement of sleep. The results were inconclusive for psychosocial distress and there was no improvement in fatigue [6]. The authors noted a high acceptability of mHealth interventions in older patients equal to that of younger populations, again challenging perceptions that mHealth is less likely to be used by older populations [13].

Kim et al [9] reviewed 37 studies from 2000 up to 2014 to assess the characteristics of web-based SMS interventions in heterogeneous populations in survivors of cancer and to perform a meta-analysis to assess the effect of these interventions. The results indicated that automated and communicative functions were the most popular mode of intervention, where the former produced automated messages and feedback for patients, while the latter allowed patients to communicate to health care workers to receive advice. The effects on diverse outcome measures including fatigue, depression, anxiety, and overall quality of life were small to moderate [9].

Singleton et al [10] reviewed 32 papers to evaluate the effectiveness of digital interventions on patient-reported outcomes (quality of life, self-efficacy, and mental or physical health) in patients who were undergoing breast cancer treatment and in patients who completed breast cancer treatment. The results revealed a significant improvement in quality of life, self-efficacy, and fatigue. The moderator analysis revealed improved quality of life for patients with cancer undergoing treatment compared to patients with cancer after active treatment. Their analysis also revealed that age was not a significant moderator for quality of life, self-efficacy, and mental or physical health [10].

Buneviciene et al [8] reviewed 25 papers to evaluate the impact of mHealth interventions in optimizing the health-related quality of life of patients with cancer. They identified that physical activity or fitness interventions, cognitive behavioral therapy, and mindfulness or stress management were the most commonly studied interventions [8].

Sarbaz et al [11] reviewed 19 papers to evaluate the effect of mHealth interventions in the management of chemotherapy-induced side effects among patients with cancer. They identified that mHealth interventions were capable of producing significant improvement in patients’ quality of life and patient satisfaction [11].

Luo et al [12] reviewed 24 papers in a meta-analysis to determine the effectiveness of mHealth-based SM interventions on medical, behavioral, and emotional management in patients with breast cancer. They identified that the interventions can potentially facilitate management and health-related quality of life (functional exercise compliance, self-efficacy, and lymphedema reduction) in patients with breast cancer [12].

To address these gaps, this systematic review aimed to update the evidence with a focus on the impact of digital technology on building SM core skills in patients with cancer. Specifically, the review’s objectives were to (1) examine what were the SM core skills that digital interventions enable and support and (2) identify any predictors of the effect of using digital health intervention on SM core skills such as age, sex, and socioeconomic status.

## Methods

This systematic review was performed according to the PRISMA (Preferred Reporting Items for Systematic Reviews and Meta-Analyses) guidelines, which is described in Multimedia Appendix 1. This review has been registered in the International Prospective Register of Systematic Reviews (PROSPERO; CRD42021221922).

Studies were included if they included participants of any age diagnosed with any type of cancer. The studies had to be randomized controlled trials (RCTs), involving 1 or more digital health technology interventions, published between January 2010 and February 2022, and written in English. The timeframe of 12 years was applied as it coincides with the emergence of research into using digital technology as SMS in the care of patients with cancer [14].

The RCTs needed to compare at least 1 digital technology intervention used to enable SM and SMS of cancer to a control that did not use technology. The design of the study had to measure a change from baseline to postintervention in 1 or more of the 6 SM core skills: problem-solving, decision-making, behavioral self-monitoring and tailoring, setting goals, partnering with health care providers, and risk reduction [1]. The study outcomes of the RCTs needed to explicitly state that there was an investigation of SM core skills or inference could be made that SM core skills were investigated. Papers have been selected based on whether the study outcome matched the definition of any of the 6 SM core skills [1,15].

Studies were selected by searching MEDLINE, CINAHL, and Scopus using the search strategies in Multimedia Appendix 2. This search was performed by 1 author with the aid of a librarian on each of the 3 databases, using search terms related to (1) SM, (2) digital health, (3) cancer, and (4) terms of exclusion. In selecting papers for inclusion in the review, the study needed to investigate the impact of their digital technology intervention on a sample of patients with cancer and measure study outcomes that matched the definition of the SM core skills. When the study did not have features that met these criteria, the study was excluded from the review.

The search results were managed using Covidence (Veritas Health Innovation Ltd) and duplicates were removed. Two reviewers were involved in doing the data extraction independently through Covidence. Each reviewer independently assessed the titles and abstracts against the eligibility criteria. Any disagreements in study selection between the 2 reviewers were resolved through discussion to produce a filtered list for further full-text review. This was followed by a full-text review considered against the eligibility criteria followed by a further discussion to resolve disagreements and to produce a final list of studies for inclusion into this systematic review.

A narrative synthesis of the results was used to assess the aggregate extracted data on digital technology intervention, outcomes, and predictors of outcomes. This approach was selected due to the heterogeneity in the intervention provided by outcomes found.

The quality of the studies was assessed through the Manual for Quality Scoring of Quantitative Studies [16] by the same 2 independent reviewers who performed the data extraction.

## Results

Data extraction is summarized in the PRISMA diagram (Multimedia Appendix 3). A search on MEDLINE, Scopus, and CINAHL on March 5, 2022, yielded 2454 studies, of which 1526 (62.2%) studies were selected after removing duplicates. Of these 1526 studies, 246 (16.1%) studies were selected as they met the eligibility criteria after abstract assessment. A further full-text screening was completed for these 246 studies and found that 12 (4.9%) papers reporting on RCTs met the eligibility criteria (Multimedia Appendix 3). A summary of the 12 papers are reported in Multimedia Appendix 4 [17-28].

A total of 4 studies were conducted in the United States [17-20]; 4 were conducted in the Netherlands [21-24]; and there were single studies from Ireland [25], Finland [26], Sweden [27], and China [28]. The most common digital technology studied were web-based applications (8 studies); with single studies examining a combined website and text messaging intervention, text messaging, educational videos, and mobile phone apps. Participants in 7 studies included female participants with breast cancer while the remaining 5 studies included both sexes, with 1 including patients with lymphoma and the remainder including multiple cancer types. In the 12 selected studies, there were 2627 participants in total, where 2143 (81.6%) participants were female and the remaining were male.

The quality assessment scores, using the Manual for Quality Scoring of Quantitative Studies [16], indicated that all studies were of high quality. The summary score of the studies ranged from 19 to 26 points out of 28 points, and the median score was 22 (IQR 2) points. The main reason that the median score was lower than the maximum score was the lack of blinding (9 studies) [17-19,21-25,29,30] due to the nature of the study. A total of 5 studies did not define their outcome variables [22-26]. Three studies had no evidence of consideration of controlling confounding variables [18,19,22] and 2 studies had incomplete control of confounding [17,21].

In assessing the 12 papers, the interventions were analyzed to identify which SM core skills (problem-solving, decision-making, behavioral self-monitoring and tailoring, setting goals, partnering with health care providers, and risk reduction) were being introduced. Across the 12 studies examined, the median number of SM core skills targeted by the interventions was 3 (IQR 2) SM core skills ranging from 1 to 5 SM core skills. A total of 11 studies explicitly stated the SM core skills that were targeted by the intervention.

The most common SM core skill targeted that were explicitly written were partnering with health care professionals [17,22,23,25,27,28] followed by behavioral self-monitoring and tailoring [17,20,23,24] and decision-making [20,24,26,27]. In 7 RCTs, additional SM core skills were identified that were not explicitly named in the study methodology. In these 7 RCTs, the most common SM core skills targeted were decision-making [17,18,23,28] and goal setting [18,19,22,28]. Overall, the most common SM core skills either explicitly identified or inferred by the reviewers were decision-making [17,18,20,23,24,26-28] and goal setting [18-20,22,25,28].

A total of 4 RCTs used a theoretical basis for intervention development including self-determination theory [25], Lazarus and Folkman’s [31] stress and coping conceptual method [19], empowering patient education theory [26], and Bandura’s [32] self-efficacy theory with self-exchange theory [28]. Furthermore, 3 RCTs used input from health care professionals [21,23,27] and 2 RCTs used problem-solving–based protocols [22,24], with 3 RCTs not stating their basis for intervention development [17,18,20].

A total of 8 (67%) out of 12 RCTs demonstrated statistically significant improvement in outcomes including self-efficacy [17,20,22,28], survivorship care knowledge and attitude [18], quality of life [25], increased knowledge of treatment [26], and emotional and social functioning [24]. In these 8 studies, the most common SM skills targeted were decision-making [17,18,20,24,26,28], followed by goal setting [18,20,22,25,28] and partnering with health care professionals [17,18,22,25,28]. A total of 5 (62%) out of 8 positive RCTs used theoretical considerations in their study design [22,24-26,28] whereas 1 (25%) out of 4 negative RCTs used theoretical considerations to design the intervention [19].

Out of the 8 papers that showed improved outcomes with the digital technology intervention, 3 RCTs investigated predictors of effects. In these 3 papers, there were 109 (16.2%) male participants out of 673 total participants. Willems et al [33] had the greatest number of male participants with 93 (25.2%) out of 369 participants, while Siekkinen et al [26] had the least, with 16 (9.1%) male participants out of 176 participants.

Siekkinen et al [26] investigated how a web-based application that gave participants feedback after responding to a knowledge test on radiotherapy increased their knowledge, leading to improved decision-making skills in patients with breast cancer. A significant positive association was observed with younger age and baseline decision-making skills (regression coefficient [RC]=–0.06, 95% CI –0.10 to –0.02; *P*=.002). A significant positive association was also observed between computer literacy and an increase in decision-making skills (RC=–0.20, 95% CI –0.37 to –0.03; *P*=.03).

Willems et al [33] evaluated the short-term effectiveness of a web-based psychoeducational program for survivors of cancer. Patients who were male (SD 0.34 in social functioning; *P*=.009); had higher education (SD 0.19 in social functioning; *P*=.04); aged 56 years and younger (SD 0.44 in fatigue; *P*<.001); or received chemotherapy with or without surgery compared to participants who received surgery only, radiotherapy with or without surgery, or chemotherapy and radiotherapy with or without surgery (SD 0.36 in depression; *P*=.008) showed higher improvement in the following SM core skills: problem-solving, behavioral self-monitoring and tailoring, goal setting and risk reduction, and decision-making skills.

Leach et al [20] assessed the efficacy of a web and text message support application for patients with cancer in managing issues related to long-term and late effects of cancer treatment. There was a statistically significant difference in developing the following SM core skills—setting goals, decision-making, behavioral self-monitoring, and completed cancer treatment (Cohen *d* for self-efficacy in patients that completed cancer treatment=0.31; *P*=.02), but there was no association observed with age (Cohen *d* for self-efficacy in participants over 60 years old=0.25; Cohen *d* for participants under 60 years old=0.29).

## Discussion

### Principal Findings

This systematic review sought to understand how digital interventions improved SM in cancer by examining what were the SM core skills that digital interventions enabled and supported and any predictors of effect. The review demonstrated that digital technology was associated with improvements in multiple SM skills; however, no study targeted all SM core skills.

The most common SM core skills targeted and improved by the interventions (both explicitly and inferred) were decision-making, followed by goal setting and partnering with health care professionals. In 8 studies that had shown improvement in outcomes, decision-making was the most common SM skill that was targeted, suggesting the importance of this skill in the overall SM process. This is consistent with findings from the systematic reviews that focused on the impact of digital technology on patients with a variety of other chronic diseases [34,35]. Future digital technology interventions should target decision-making, goal setting, and partnering with health care professionals to improve outcomes for patients with cancer given this supportive evidence.

In comparison, fewer studies targeted and observed an improvement in problem-solving [21,24,26,28], behavioral self-monitoring and tailoring [17,20], and risk reduction [24]. These findings imply that there is potential from all 6 SM core skills to build SM through mHealth interventions.

In line with the Corbin and Strauss [36] framework, chronic disease management, including cancer, requires the patient to address 3 distinctive tasks: medical management of their condition such as taking medication or responding to symptoms, managing behaviors and life roles, and dealing with emotional consequences of the illness. These tasks call for the use of diverse skills in the context of how the patient perceives their circumstances and problems. This process of SM can be supported by health system interventions designed to deliver SMS. It is thus perhaps not surprising that the most commonly targeted skills in our study included decision-making, goal setting, and partnering with health professionals.

It Is a little surprising and concerning that problem-solving was not as frequently targeted, given that the nature of SM is addressing problems as defined by patients. It is notable that decision-making contributes to problem-solving with the latter also including the identification of problems, the generation of solutions, and their implementation and evaluation. This lesser attention to problem-solving and similar lesser focus on behavioral self-monitoring and risk reduction may reflect the prevalent medical approach to chronic disease management where the delivery of solutions and interventions rests within the health system rather than the patient. Future research should explore the reasons for less focus on some of the SM skills from the perspective of the patients and the health professionals alike. Furthermore, the design of future interventions should consider the key SM skills required for particular interventions from the users’ perspective and ensure that they are adequately supported in the interventions.

This study highlights a number of gaps in the design of studies focusing on SMS of patients with cancer, which are often not grounded in theory and not taking a systematic approach to the “active ingredient” of SM, that is, the core skills that patients use. It is impossible to accurately state why these deficiencies exist in the first place, but they point to some potential strategies to avoid them in future studies, including the support of theory-driven research in SM and consistent standards of reporting of these types of studies.

None of the RCTs in this review targeted all SM core skills. An average of 3 SM core skills out of the total 6 SM core skills were targeted. Lorig and Holman’s [15] review of 38 RCTs that have incorporated 1 or more SM core skills as interventions for participants with chronic diseases proposed that there was greater potential in assisting patients with chronic diseases to become self-managers of their disease by using all SM core skills to promote behaviors for good medical management, emotional management, and role management. It is likely that targeting more SM core skills would be beneficial in drawing on a range of skills to manage different tasks involved with cancer management. However, different tasks may not require all the SM core skills for optimal health outcomes.

Future research on digital interventions should target multiple SM core skills explicitly and consider which SM core skills are most required to improve the SM of cancer. A greater understanding and use of behavioral theoretical frameworks of SM may also assist in identifying and prioritizing SM core skills necessary for the tasks involved with cancer management. In doing so, there is potential in producing mHealth technology where patients with cancer are capable of managing their illness and reducing their risk of deterioration leading to hospitalization.

It was notable that 5 (62%) out of the 8 RCTs that used theoretical frameworks in designing an SM intervention produced statistically significant outcomes. This observation is consistent with the findings of the systematic review of SMS inventions in primary care management of chronic diseases by Dineen-Griffin and colleagues [37], who showed that theoretical models produced effective frameworks in SMS and improvements were seen in clinical indicators, health-related quality of life, confidence to self-manage, disease knowledge, and control [29]. These findings emphasize the necessity of including theoretical frameworks in future digital intervention studies design.

Only 3 (25%) [20,26,33] out of 12 studies explored predictors of effect and concluded that younger age, male sex, higher education, computer literacy, completing cancer treatment, and being a recipient of chemotherapy were associated with improving the development of SM core skills. Excluding cancer treatment, each factor was not identified by more than 1 paper as a predictor of effect. As of now, previous reviews have investigated the predictors of the use of eHealth on patients with chronic diseases, showing that younger age was associated with higher eHealth use but there were inconsistent results with regards to sex and education [38,39]. Within the 3 papers, only Leach et al [20] investigated and identified age as an association with building SM core skills for patients with cancer. These findings may suggest that they may play some role in the predictor of effects but there is limited evidence from the current findings of this paper to support this as of now.

Within this systematic review, there was sex bias where 81.6% (2143/2627) of the total participants in the 12 selected studies were female. Within the studies that investigated the predictors of effect, 85.6% (656/766) of the total participants were female. This raises a possibility of limitations to the extent that sex could be a predictor of effect in building SM skills with mHealth interventions. Further research on the impact of predictors of digital intervention effect including sex is needed. There is relatively limited data on specific predictors of intervention effectiveness such as sex and age, with few studies and small patient numbers addressing this issue. Future research should focus on robust examination of predictors of intervention effectiveness.

The study population in 7 (58%) of 12 included studies included patients with breast cancer [17,19,21,25-28], suggesting that the literature may not be representative of other cancers. In 11 (92%) of the 12 papers, the location of the studies was in North America and Europe, suggesting a lack of evidence relevant to populations from other regions and ethnicities.

It is also noteworthy that all studies identified in this review had participants from metropolitan backgrounds despite the growing importance of digital technology in patients from rural areas [40].

The majority (8/12, 67%) of the studies in this review used web-based application interventions in assessing how SM was built in patients with cancer, with less data on other modalities such as telehealth and mobile phone apps. In addition, there are other modalities of technology that have shown improvement in building SM for patients with diabetes including artificial intelligence (AI) [41] and virtual reality applications [42]. There is a growing interest in AI and machine learning as an approach to deliver coaching-like approach to improve behaviors, especially with regards to physical activity, mental well-being, decision-making, and problem-solving, although the data on the mechanisms of how these approaches improve SM skills remain, as yet, limited. Greater adoption of machine learning approaches will likely facilitate greater customization and tailoring of interventions, integration into overall care, and focus on specific patient subpopulations. It is critical that the design process for such interventions is based on sound behavioral models, and factors in consideration of customization, behavioral change, and self-efficacy in its evaluation [43].

### Conclusions

Digital technology appears to improve SM core skills including decision-making, goal setting, and partnering with health care partners in patients with cancer with suggestion of greater impact in people who are younger, male, educated, highly computer literate, completing cancer treatment, and a recipient of chemotherapy. These findings should prompt developers or designers of digital health intervention to focus on interventions targeting multiple SM core skills and better identifying predictors of digital intervention effect.

## Appendix

Multimedia Appendix 1 PRISMA (Preferred Reporting Items for Systematic Reviews and Meta-Analyses) checklist.

Multimedia Appendix 2 Search strategies used for CINAHL, MEDLINE, and Scopus.

Multimedia Appendix 3 PRISMA (Preferred Reporting Items for Systematic Reviews and Meta-Analyses) diagram summarizing the searched literature.

Multimedia Appendix 4 Summary of the 12 papers that describes the impact of digital technology on building self-management skills for patients with cancer.

## Abbreviations

AI: artificial intelligence
mHealth: mobile health
PRISMA: Preferred Reporting Items for Systematic Reviews and Meta-Analyses
PROSPERO: International Prospective Register of Systematic Reviews
RC: regression coefficient
RCT: randomized controlled trial
SM: self-management
SMS: self-management support
