# Supplementary material for: The Impact of Digital Technology on Self-Management in Cancer: Systematic Review
Source: JMIR Cancer. 2023 Nov 22;9:e45145. doi: 10.2196/45145 (PMC10701654; doi:10.2196/45145)
Supplement: Multimedia Appendix 2 [file cancer_v9i1e45145_app2.docx]

**Search terms for Medline**

1     exp cancer/ (3365081)
2     exp neoplasms/ (3365081)
3     (cancer* or neoplasm* or tumo?r* or carcinoma* or sarcoma* or adenocardinoma* or adeno?carcinoma* or
adenoma*).mp,kw. (4146732)
4     1 or 2 or 3 (4527743)
5     exp telemedicine/ (29983)
6     exp cellular phone/ (10887)
7     exp text messaging/ (3003)
8     (eHealth or e-Health).mp. (7014)
9     (mHealth or m-health or mobile health).mp. (11447)
10     (telehealth or tele-health).mp. (5843)
11     (telemedicine or tele-medicine).mp. (30487)
12     (telecare or telehealthcare or telehomecare or mobile telehealthcare or mobile telemedicine or mCare or
m-care).mp. (1033)
13     Telecommunications.mp. (6686)
14     (telepharmacy or tele-pharmacy).mp. (98)
15     telemonitor*.mp. (1684)
16     telemanagement*.mp. (69)
17     e-compliance*.mp. (8)
18     (mobile applications adj2 (phone* or mobile* or device*)).mp. (7357)
19     (mobile communication or mobile technolog* or mobile devic*).mp. (5860)
20     Computers/ or Microcomputers/ or Internet/ (134147)
21     Text Messaging/ or (texting or text messag* or messag* or text* or short message or SMS).mp. (226121)
22     (smartphone or smart-phone).mp. (12124)
23     GPRS.mp. (245)
24     Wireless.mp. (15773)
25     bluetooth.mp. (1207)
26     5 or 6 or 7 or 8 or 9 or 10 or 11 or 12 or 13 or 14 or 15 or 16 or 17 or 18 or 19 or 20 or 21 or 22 or 23 or 24
or 25 (430199)
27     "self management support".mp,kw. (1221)
28     (self adj2 (care or manag* or administ* or medicat* or monitor* or efficac* or guid* or regulat* or direct* or
determin*)).mp,kw. (190418)
29     (selfcar* or selfmanag* or selfadminist* or selfmedicat* or selfmonitor* or selfefficac* or selfguid* or
selfregulat* or selfdirect* or selfdetermin*).mp,kw. (402)
30     (personal adj2 manag*).mp,kw. (851)
31     ((patient? or client?) adj2 participat*).mp,kw. (45893)
32     (animals not humans).sh. (4705549)
33     (comment or editorial or meta-analysis or practice-guideline or review or letter or "systematic
review").mp,kw,pt. (5428291)
34     or/27-31 (233183)
35     4 and 26 and 34 (1286)
36     35 not (32 or 33) (1032)

**Search terms for CINAHL**

| **#** | **Query** | **Results** |
| --- | --- | --- |
| S33 | S4 AND S26 AND S32 | 1,236 |
| S32 | S27 OR S28 OR S29 OR S30 OR S31 | 156,429 |
| S31 | TX ((patient? or client?) N2 participat*) | 13,174 |
| S30 | TX (personal N2 manag*) | 1,066 |
| S29 | TX (selfcar* or selfmanag* or selfadminist* or selfmedicat* or selfmonitor* or selfefficac* or selfguid* or selfregulat* or selfdirect* or selfdetermin*) | 1,505 |
| S28 | TX (self N2 (care or manag* or administ* or medicat* or monitor* or efficac* or guid* or regulat* or direct* or determin*)) | 143,244 |
| S27 | TX "self management support" | 825 |
| S26 | S5 OR S6 OR S7 OR S8 OR S9 OR S10 OR S11 OR S12 OR S13 OR S14 OR S15 OR S16 OR S17 OR S18 OR S19 OR S20 OR S21 OR S22 OR S23 OR S24 OR S25 | 201,651 |
| S25 | TX bluetooth | 361 |
| S24 | TX wireless | 14,585 |
| S23 | TX GPRS | 238 |
| S22 | TX (smartphone or smart-phone) | 7,921 |
| S21 | MH "text messaging" OR TX ( texting or text messag* or messag* or text* or short message or SMS ) | 94,784 |
| S20 | MH computer OR MH microcomputers OR MH internet | 51,065 |
| S19 | TX (mobile communication or mobile technolog* or mobile devic*) | 5,155 |
| S18 | TX (mobile applications N2 (phone* or mobile* or device*)) | 9,326 |
| S17 | TX e-compliance | 9 |
| S16 | TX telemanagement* | 45 |
| S15 | TX telemonitor* | 875 |
| S14 | TX (telepharmacy or tele-pharmacy) | 99 |
| S13 | TX telecommunications | 4,722 |
| S12 | TX (telecare or telehealthcare or telehomecare or mobile telehealthcare or mobile telemedicine or mCare or m-care) | 2,633 |
| S11 | TX (telemedicine or tele-medicine) | 15,432 |
| S10 | TX (telehealth or tele-health) | 12,026 |
| S9 | TX (mHealth or m-health or mobile health) | 8,064 |
| S8 | TX (eHealth or e-Health) | 5,410 |
| S7 | (MH "Text Messaging+") | 3,035 |
| S6 | (MH "Cellular Phone+") | 7,331 |
| S5 | (MH "Telemedicine+") | 14,256 |
| S4 | S1 OR S2 OR S3 | 786,485 |
| S3 | TX (cancer* or neoplasm* or tumo?r* or carcinoma* or sarcoma* or adenocardinoma* or adeno?carcinoma* or adenoma*) | 735,472 |
| S2 | "cancer" | 425,229 |
| S1 | (MH "Neoplasms+") | 550,791 |

**Search terms for SCOPUS**

( TITLE-ABS -KEY ( cancer*  OR  neoplasm*  OR  tumo?r*  OR  carcinoma*  OR  sarcoma*  OR  adenocardinoma*  OR  adeno?carcinoma*  OR  adenoma*  OR  hodgkin*  OR  nonhodgkin*  OR  adenocarcinoma*  OR  leuk?emia*  OR  metasta*  OR  malignan*  OR  lymphoma*  OR  melanoma*  OR  myeloma*  OR  oncolog* ) )

AND  ( TITLE-ABS-KEY ( "eHealth"  OR  "e-Health"  OR  "mHealth"  OR  "m-health"  OR  "mobile health"  OR  "telehealth"  OR  "tele-health"  OR  "telemedicine"  OR  "tele-medicine"  OR  "telecare"  OR  "telehealthcare"  OR  "telehomecare"  OR  "mobile telehealthcare"  OR  "mobile telemedicine"  OR  "mCare"  OR  "m-care"  OR  "telecommunications"  OR  "telepharmacy"  OR  "tele-pharmacy"  OR  "telemonitor*"  OR  "telemanagement"  OR  "e-compliance"  OR  ( "mobile applications"  W/2  ( "phone*"  OR  "mobile*"  OR  "device*" ) )  OR  "mobile communication"  OR  "mobile technolog*"  OR  "mobile devic*"  OR  "computers"  OR  "microcomputers"  OR  "internet"  OR  "text messaging"  OR  "texting"  OR  "text messag*"  OR  "messag*"  OR  "text*"  OR  "short message"  OR  "SMS"  OR  "smartphone"  OR  "smart-phone"  OR  "GPRS"  OR  "wireless"  OR  "bluetooth" ) )

AND  ( TITLE-ABS-KEY ( "self management support"  OR  "self care"  OR  "self-management"  OR  self  W/2  ( care  OR  manag*  OR  administ*  OR  medicat*  OR  monitor*  OR  efficac*  OR  guid*  OR  regulat*  OR  direct*  OR  determin* )  OR  ( selfcar*  OR  selfmanag*  OR  selfadminist*  OR  selfmedicat*  OR  selfmonitor*  OR  selfefficac*  OR  selfguid*  OR  selfregulat*  OR  selfdirect*  OR  selfdetermin* )  OR  ( personal  W/2  manag* )  OR  ( ( patient?  OR  client? )  W/2  participat* ) ) )

AND NOT  ( ( TITLE-ABS-KEY ( animal*  AND NOT  human ) )  OR  ( TITLE-ABS-KEY ( meta-analy*  OR  metaanaly*  OR  review ) ) )  AND  ( LIMIT-TO ( PUBYEAR ,  2020 )  OR  LIMIT-TO ( PUBYEAR ,  2019 )  OR  LIMIT-TO ( PUBYEAR ,  2018 )  OR  LIMIT-TO ( PUBYEAR ,  2017 )  OR  LIMIT-TO ( PUBYEAR ,  2016 )  OR  LIMIT-TO ( PUBYEAR ,  2015 )  OR  LIMIT-TO ( PUBYEAR ,  2014 )  OR  LIMIT-TO ( PUBYEAR ,  2013 )  OR  LIMIT-TO ( PUBYEAR ,  2012 )  OR  LIMIT-TO ( PUBYEAR ,  2011 )  OR  LIMIT-TO ( PUBYEAR ,  2010 ) )
