# Supplementary figures and images for: The Impact of Digital Technology on Self-Management in Cancer: Systematic Review
Source: JMIR Cancer. 2023 Nov 22;9:e45145. doi: 10.2196/45145 (PMC10701654; doi:10.2196/45145)

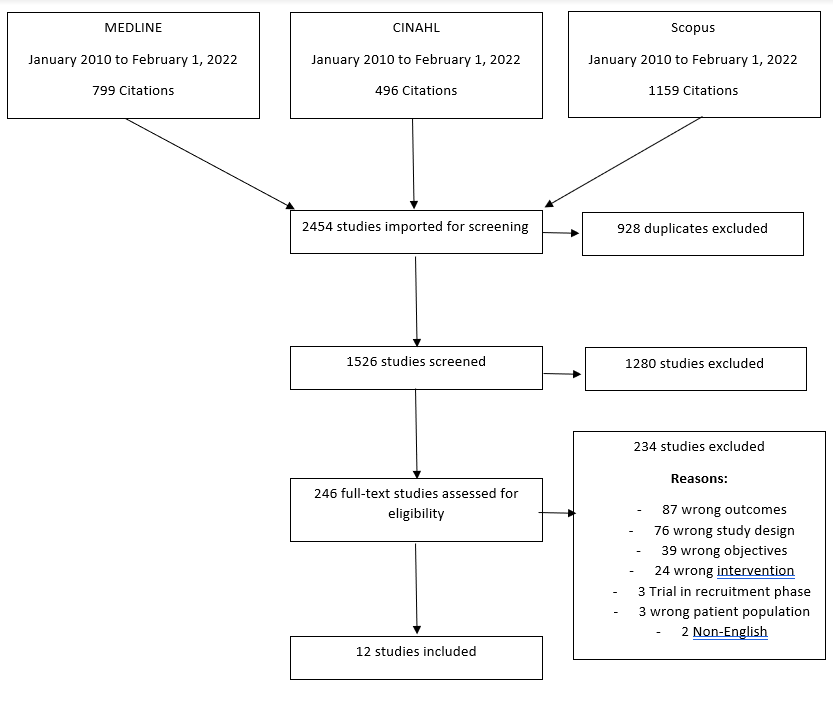


**Figure S1: PRISMA Diagram summarising the searched literature**

Supplement: Multimedia Appendix 3 [file cancer_v9i1e45145_app3.docx]
