# Supplementary material for: The Impact of Digital Technology on Self-Management in Cancer: Systematic Review
Source: JMIR Cancer. 2023 Nov 22;9:e45145. doi: 10.2196/45145 (PMC10701654; doi:10.2196/45145)
Supplement: Multimedia Appendix 4 [file cancer_v9i1e45145_app4.docx]

**Table S1. Studies that demonstrated statistically significant improvements in study outcomes.**

| **Author, year, location** | **Objectives** | **Intervention** | **Basis of Digital Intervention Design** | **Study Population** | **Outcomes measured** | **Results** | **SM core skills explicitly targeted in the study** | **Other SM core skills as inferred by reviewers** | **Predictors of outcomes** | **Quality Assessment score** |
| --- | --- | --- | --- | --- | --- | --- | --- | --- | --- | --- |
| Borosund 2014, USA | To compare the effects of an internet-based patient provider communication service (IPPC) and WebChoice, a web based illness management system in the self-management of patients and usual care on symptom distress, anxiety, depression and self efficacy. | IPPC: an online service to ask questions, share experiences with or get advice from oncology nurses.  WebChoice: Access to reliable web sources and SM activities. | Not Stated | Patients, over the age of 18, with a recent diagnosis of breast cancer treated with surgery or under treatment with radiation, chemotherapy, hormone therapy or combination of those, having access to Internet at home. n= 167.  Number of female participants = 167. | Primary outcomes: Symptom distress, anxiety and depression  Secondary outcomes: Self-efficacy | No significant differences in self-efficacy were found between the IPPC group and the usual care group.  The Webchoice group tended to score higher than the usual care group on self-efficacy (Mean difference = 8.81, *P* = .08). | 1) Behavioral self-monitoring and tailoring  2) Partnering with Health-care providers | 1) Decision making  2) Risk Reduction & Health Maintenance | Not studied. | 21/28 |
| Casillas 2020, USA | To assess the efficacy of text-messaging and peer navigation interventions to increase adolescent and young adult late effects knowledge and knowledge, attitudes, and self-efficacy towards seeking survivor-focused care. | Text-messaging- allows access to community and cancer center resources | Not Stated | Patients, 15-39 years old, who have received surgery, chemotherapy or radiation for their cancer treatment, off cancer treatment for more than a year and possessed a personal cell phone with text-messaging capabilities. n = 78.  Number of female participants = 37. Number of male participants = 41. | Primary Outcomes: Survivorship care knowledge, survivorship care attitude, survivorship self-efficacy assessment | The text-messaging group had increased survivorship care knowledge and late effects knowledge compared to the control group (Effect Size = 0.70, P < 0.05). There is increased attitudes towards seeking survivor-focused care in the text-messaging group compared to the control group (Mean difference = 0.33, *P* < 0.05.) | Not explicitly mentioned. | 1) Decision Making  2) Setting goals and action planning  3) Partnering with health-care providers | Not studied. | 24/28 |
| Hawkins, 2010, Ireland | To test whether three mediating process of Self-Determination Theory are involved in intervention effects on quality of life through the web-based application CHESS | CHESS:  1) Providing tailored education  2) Providing discussion groups  3) Coaching | Self-Determination theory | Female patients diagnosed with breast cancer within two last months. n = 434  Number of female participants = 434. | Quality of Life, Autonomy, Competence and Relatedness. | Patients with access to CHESS and phone conversations had higher quality of life scores than those in the other three conditions (Effect Size = 3.01, *P* <0.05). | 1) Setting goals and action planning  2) Partnering with health-care providers | None. | Not studied. | 22/28 |
| Leach 2021, USA | To compare self-efficacy for managing cancer-related issues among cancer survivors randomized to receive the enhanced Springboard Beyond Cancer intervention. | Springboard Beyond Cancer:  1) Website  2) Text-messaging | Not Stated | Patients with a history of cancer.  N = 88.  Number of female participants = 81.  Number of male participants = 7. | Self-efficacy for managing cancer. | The intervention arm increased significantly in self-efficacy for managing cancer between baseline and 3 month follow up compared to the control group. (Cohen’s d= 0.31, P =0.02) | 1) Setting goals and action planning  2) Decision making  3) Behavioral self-monitoring and tailoring | None. | No statistically significant difference was noted by age over 60 years old (Cohen’s d = 0.25, p= 0.10) and below 60 years old (Cohen’s d = 0.29, p=0.13).  A statistically significant difference was noted between those who had completed treatment (Cohen’s d = 0.31, p= 0.02) and people who were in the middle of treatment (Cohen’s d =0.11, p=0.66). | 23/28 |
| Siekkinen, 2015, Finland | To evaluate how an web e-feedback knowledge intervention (e-Re-Know), before first radiotherapy, improves breast cancer patients' knowledge of radiotherapy. | E-Re-Know Knowledge clarification via feedback | Empowering patient education theory | Women, aged 18-75 years old, with breast cancer undergoing radiotherapy at an outpatient clinic for the first time. n=128.  Number of female participants = 128. | Increase in knowledge level of radiotherapy | The E-Re-Know intervention group has a significant increase in the change of knowledge level (2.5 points, P<0.0001) | 1) Decision-making | 1) Problem solving | Younger patients and patients who had higher computer literacy had higher change in knowledge level. | 19/28 |
| Van Bruinessen, 2016, Netherlands | To evaluate if and in what way patients benefit from the web-based intervention PatientTIME and if it enhances their confidence in clinical communication. | PatientTIME intervention: 1) Video resources to tackle communication barriers with HCP 2) Playback audio of consultation | Self-developed intervention mapping framework based on modelling, tailoring information, previsit goal settings and listening to visit recordings. | 18 years old and above, had been diagnosed with malignant lymphoma, had at least one (follow-up) consultation with their healthcare professional (HCP) per year and were receiving treatment or follow up care in a hospital. n=87.  Number of female participants = 53.  Number of male participants = 34. | Perceived efficacy | More than half of the intervention group patients reported that the intervention helped them prepare for a clinical consultation; it created awareness about the importance of communication and reinforced their existing communication skills.  The intervention group showed a significant improvement in perceived efficacy (-1.97 points, p = 0.02). | 1) Partnering with health-care providers | 1) Behavioral self-monitoring and tailoring  2) Setting goals and action planning | Not studied. | 22/28 |
| Willems, Netherlands, 2016 | To evaluate the six months effects of a web-based computer tailored application in increasing emotional and social functioning and reducing depression and fatigue hold at 12 months from baseline. | KNW: eight focused modules based on the principles of problem-solving and cognitive behavioral therapy | Bartholomew’s Intervention mapping protocol | Patients diagnosed with any type of cancer, aged 18 years or older; primary treatment (surgery, chemotherapy, and/or chemotherapy) had been completed successfully for at least 4 weeks. n=462.  Number of female participants = 369.  Number of male participants = 93. | Emotional and social functioning, depression and fatigue. | The intervention was effective in reducing depression and fatigue. In addition, effects were found for emotional and social functioning. | 1) Problem Solving  2) Behavioral self-monitoring and tailoring  3) Setting goals and action planning  4) Risk reduction and health maintenance  5) Decision Making | None | 1) Being male or having higher educational levels improved social functioning.  2)Younger participants aged 56 or lower had improved fatigue levels compared to those older than 57.  3)Treatment type moderated the 6 month effect of depression. | 23/28 |
| Zhu, China, 2018 | To determine the effectiveness of a mobile application breast cancer e-support (BCS) program to address women's self-efficacy, social support, symptom distress, quality of life, anxiety and depression. | BCS: 1) Use of forums to communicate with doctors and learn about cancer 2) Educational videos | Bandura’s self-efficacy theory and self-exchange theory | Women diagnosed with any stage of breast cancer and were commencing chemotherapy. n=114.  Number of female participants = 114. | Self-efficacy, social support, symptom distress, quality of life, anxiety and depression. | Breast cancer e-support + care as usual participants had significant better health outcomes at 3 months regarding self-efficacy (21.05; 95% CI 1.87-40.22; P=.03; d=0.53), symptom interference (−0.73; 95% CI −1.35 to −.11; P=.02; d=−0.51), and quality of life (6.64; 95% CI 0.77-12.50; P=.03, d=0.46). | 1) Partnering with health-care providers | 1) Problem Solving  2) Decision Making  3) Setting goals and action planning | Not studied. | 22/28 |

**Table S2. Studies that did not demonstrate statistically significant improvements in study outcomes.**

| **Author, year, location** | **Objectives** | **Intervention**  **vs comparator** | **Basis of Digital Intervention Design** | **Study Population** | **Outcomes measured** | **Results** | **SM core skills explicitly targeted in the study** | **Other SM core skills as inferred by reviewers** | **Quality Assessment score** |
| --- | --- | --- | --- | --- | --- | --- | --- | --- | --- |
| Admiraal 2017, Netherlands | Examine the effectiveness of a web-based tailored psychoeducational program (ENCOURAGE) to empower patients to take control over prevailing psychosocial and physical symptoms. | ENCOURAGE program – a web-based program that offers  1) Problem orientation  2) Tailored psychoeducation  Control  Regular visits to medical specialist with no intervention given | Scientific literature and input from psychologists, doctors and nurses, pastoral worker and a patient advocate. | 18 years and older female patients diagnosed with primary breast cancer who completed curative-intent primary treatment within the past 6 months and have access to internet.  n = 138.  Number of female participants = 138. | Primary Outcomes: Optimism and Control over the future  Secondary Outcomes: Distress, Distress-Related Problems and Quality of Life | No differences between the intervention and control group in primary and secondary outcomes. | 1) Problem Solving | None | 21/28 |
| Ridner, 2019, USA | To evaluate the effects of a Web-based Multimedia Intervention (WBMI) for breast cancer-related lymphedema (BCRL) patients on symptom burden, function, psychological well-being, costs and arm volume after 1 month. | WBMI - Educational videos on lymphedema | Lazarus and Folkman’s Stress and Coping conceptual method | 18 years old and above with a history of breast cancer and diagnosis of stage II lymphedema and not undergoing chemotherapy or radiation. n=80.  Number of female participants = 80. | Symptom burden, psychological well-being, function of iADLs and coping. | The WBMI provided no amount of change in self-care activities compared to the control group. | 1) Problem Solving | 1) Behavioral self-monitoring and tailoring  2) Setting goals and action planning  3) Risk reduction and health maintenance | 22/28 |
| Van der Hout, 2020, Netherlands | To assess the efficacy, reach and usage of Oncokompas, a web-based eHealth application and obtain tailored feedback with a personalised overview of supportive care options. | Oncokompass: Interactive education modules and tailored feedback. | Input from healthcare professionals, cancer survivors, managerial staff and insurance companies. | Cancer survivors, aged at least 18 years, who were diagnosed with head and neck cancer, colorectal cancer, breast cancer, Hodgkin lymphoma, or non-Hodgkin lymphoma. n=625.  Number of female participants = 316.  Number of male participants = 309. | Patient activation (knowledge, skills, and confidence for SM). | Patient activation was not significantly different between intervention and control group over time (difference at 6-month follow-up 1·7 [95% CI -0·8-4·1], p=0·41). | 1) Behavioral self-monitoring and tailoring  2) Partnering with health-care providers | 1) Decision Making  2) Risk reduction and health maintenance | 26/28 |
| Ventura, Sweden, 2017 | To evaluate the impact of a computer-based educational programme (Swedish Interactive Rehabilitation Information [SIRI]) compared to standard care on health self-efficacy and health care participation in breast cancer patients and examining whether usage could be explained by demographic, medical and psychosocial factors. | SIRI: Educational resources | Participatory design based on input from patient representatives, clinicians and a health sciences researcher. | Early-stage breast cancer patients who are scheduled for breast surgery. n=226.  Number of female participants = 226. | Self efficacy and healthcare participation | SIRI did not produce statistically significant impact on health self-efficacy, healthcare participation, anxiety or depression. | 1) Decision making  2) Risk Reduction and Health Maintenance  3) Partnering with Healthcare providers | None | 23/28 |
